# Association between physical activity and infertility: a comprehensive systematic review and meta-analysis

**DOI:** 10.1186/s12967-022-03426-3

**Published:** 2022-05-23

**Authors:** Fangfang Xie, Yanli You, Chong Guan, Yuanjia Gu, Fei Yao, Jiatuo Xu

**Affiliations:** 1 Shanghai Municipal Hospital of Traditional Chinese Medicine, Shanghai University of Traditional Chinese Medicine, No.274, Middle Zhijiang Road, Shanghai, 200071 China; 2 Shanghai University of Traditional Chinese Medicine, 1200 Cailun Road, Pudong New District, Shanghai, 201203 China; 3 Department of Traditional Chinese Medicine, ChangHai Hospital, Naval Medical University, Shanghai, China

**Keywords:** Infertility, Physical activity, Meta-analysis

## Abstract

**Background:**

Physical activity (PA) may protect against infertility by modulating the hypothalamic-pituitary–gonadal axis, thereby reducing gonadotropin levels, elevating immune function, and inhibiting inflammation and circulating sex hormones. However, whether PA reduces the risk of infertility remains largely unknown. We therefore conducted a systematic review and meta-analysis to determine the preventive effects of PA on infertility.

**Methods:**

We searched PubMed, Cochrane Library, EMBASE, and CINAHL databases to retrieve published epidemiologic studies on the relationship between PA and infertility. Following the PRISMA guidelines, we selected English literature publishedprior to 11 April 2022, and assessed study quality using the Newcastle–Ottawa Scale. Our protocol, including the full methods employed for this review, is available on PROSPERO (ID = CRD42020143344).

**Results:**

Six cohort studies and four case–control studies based on 708,965 subjects and 12,580 cases were eventually screened and retained. High levels of PA were shown to reduced risk of infertility relative to low levels (cumulative relative risk [RR] = 0.59, with a 95% confidence interval CI 0.49–0.71), and we reported results for cohort studies (RR = 0.63, 95% CI 0.50–0.79) and case–control studies (RR = 0.49, 95% CI 0.35–0.67). Our findings were comparable for men (RR = 0.65, 95% CI 0.41–1.04) and women (RR = 0.56, 95% CI 0.47–0.66). The meta-analysis of six risk estimates from five studies of low, moderate, and high PA levels showed that moderate PA may also reduce the risk of infertility compared with low PA (RR = 0.54, 95% CI 0.38–0.77). However, high PA also appeared to slightly augment the risk of infertility compared with moderate PA (RR = 1.31, 95% CI 1.08–1.59).

**Conclusions:**

This present systematic review comprehensively reflected an inverse relationship between different levels of PA and infertility, and our meta-analysis showed that a moderate-to-high PA level significantly reduced the overall risk of infertility, and that this level of PA activity was a common protective factor. In addition, limited evidence suggested that compliance with international PA guidelines would greatly lower the risk of infertility (RR = 0.58, 95% CI 0.45–0.74; I^2^ = 0.0%). Future studies, however, need to be executed to further determine the frequency, optimal dosage, and duration required to effectively attenuate the risk of infertility.

**Supplementary Information:**

The online version contains supplementary material available at 10.1186/s12967-022-03426-3.

## Background

Infertility is a disease characterized by failure to confirm clinical pregnancy after 12 months of routine unprotected intercourse, or due to the impaired reproductive capability of the individual or their partner [1]. The prevalence rate in more developed nations is 3.5% to 16.7%, and that in less developed nations is 6.9% to 19.3%, with the overall median prevalence estimated to be 9% [2]. The incidence of infertility has increased significantly, and it has become the third most serious disease after cancer and cardiovascular disease [3]; it has been estimated that 186 million people worldwide are affected by infertility, the majority of whom are residents of developing countries [4, 5]. Infertility exerts a negative impact on overall health, population numbers, and socio-economic factors [6], and the inability to produce children affects men and women worldwide. Infertility can cause depression, pain, loss of control, low self-esteem, marital distress, and sexual dissatisfaction—as well as societal discrimination and ostracism [7, 8]. The exact cause of infertility remains unknown, although the morbidity associated with this condition is high. Several studies have suggested that late childbirth, illegal and legal abortions, genetic variation, and the excessive use of contraception are all possible factors involved in the growth of infertility [6, 9–11].

Both psychotherapy and pharmacotherapy are effective treatments for infertility. Medications for female infertility such as clomiphene citrate have developed into first-line treatments for infertility and are gaining popularity among the general public. However, its side effects include—but are not limited to—insomnia, headache, mood swings, dizziness, hair loss, visual disturbances, and multiple pregnancy [12]. Although cognitive behavior therapy (CBT) is an alternative to pharmacotherapy that aims to diminish anxiety and to promote the mental health of infertile women, few authors have assessed the effectiveness of CBT interventions in the field of infertility [13, 14]. Therefore, the prevention of infertility will avoid the long, expensive, and difficult processes involved in pregnancy.

One potential candidate that is increasingly being used to treat infertility is physical activity (PA). The World Health Organization (WHO) defines PA as "any physical movement produced by skeletal muscle that consumes energy" [15], and recommends more than 150 min of high PA per week to reduce the risk of reproduction [16]. When properly prescribed, PA is an inexpensive and universal “medication” with minimal side effects; PA is a veritable "home pharmacy" that we always carry with us [17]. PA appears to reduce infertility through biologic and physiologic mechanisms by strengthening antioxidant defenses and reducing inflammation of bodily fluids, organs, and tissues [18, 19]. Some randomized controlled trials have reported a therapeutic effect of PA on infertility that acts through systemic effects such as increased immune function, insulin resistance, and circulating sex hormones [20]. However, in contrast to the well-known beneficial effects of regular PA on many adverse health outcomes (including prevention of premature death), the effect of PA on fertility in the general population is unclear [21].

A large number of meta-analyses in recent years have revealed that PA reduces the risk of endometriosis in infertile women, while others suggest that PA in polycystic ovary syndrome improves reproductive performance [22]. However, to our knowledge, few meta-analyses have been undertaken to evaluate the effects of PA on infertility. Therefore, in the present systematic review and meta-analysis we assessed whether the implementation of PA reduced the risk of infertility. In addition, equally important potential regulatory factors and their relationships to infertility including alcohol consumption, smoking, and household income factors were also evaluated. Although we still recognize very little in terms of the duration and intensity of PA in reducing the risk of infertility, our results are likely to provide valuable information with respect to clinical guidelines and interventions in refining the design of primary interventions in infertility.

## Main text

This systematic review and meta-analysis followed the Preferred Reporting Items for Systematic Reviews and Meta-analyses (PRISMA) guidelines, and our protocol, including the full methods for this review, is available on https://www.crd.york.ac.uk/PROSPERO/#recordDetails (ID = CRD42020143344) (Additional file 2).

### Literature search

We evaluated relevant studies publishedto 11 April 2022 in the EMBASE, Cochrane Library, PubMed, and CINAHL databases. We used the OR operator to connect these descriptors of PA: ‘physical activity’, ‘Exercises’, ‘Activities, Physical’, ‘Activity, Physical’, ‘Physical Activities’, ‘Exercise, Physical’, ‘Exercises, Physical’, ‘Physical Exercise’, ‘Physical Exercises’, ‘Acute Exercise’, ‘Acute Exercises’, ‘Exercise, Acute’, ‘Exercises, Acute’, ‘Exercise, Isometric’, ‘Exercises, Isometric’, ‘Isometric Exercises’, ‘Isometric Exercise’, ‘Exercise, Aerobic’, ‘Aerobic Exercise’, ‘Aerobic Exercises’, ‘Exercises, Aerobic’, ‘Exercise Training’, ‘Exercise Trainings’, ‘Training, Exercise’, and “Trainings, Exercise”. Subsequently, we employed an AND operator to combine the previous terms with the following terms for infertility outcomes: “Infertility”, “Sterility, Reproductive”, “Sterility”, “Subfertility”, and “Sub-Fertility”. Our search strategy was based upon human-oriented research articles in English, and we also examined reference lists that met the search strategy so as to allow searching for other pertinent studies.

### Inclusion and exclusion criteria

Two authors (F.F.X. and C.G.) comprehensively and independently reviewed the articles. Studies were included if they: (1) investigated the relationship between PA and infertility; (2) used observational studies in cohort or case–control designs; (3) published odds ratios (OR), relative risks (RR), or hazard ratios (HR); and (4) reported a relative risk with its corresponding 95% confidence interval (CI) or sufficient information to calculate these indices. We excluded studies that combined PA with other interventions such as diet, behavioral therapy, or antidepressant medication; and also excluded those that were classified as an editorial, review, meta-analysis, comment, news, letter, or practice guideline Additional file 1.

### Data extraction

All retrieved studies were independently screened by two individuals, and if a paper was considered relevant, it was included and a standardized qualification form was used to retrieve the complete article and potentially related research that allowed us to conduct a detailed assessment. If the reviewer disagreed with the qualifications of the study, an agreement was reached after discussion.

To assess the potential differences in PA relative to infertility, we extracted the first author’s last name; number of cases; publication date; sex; sample size; study design and location; definition of highest, moderate, and lowest levels of physical activity; relative risk estimates with corresponding 95% confidence intervals (CI); and adjustment factors. We extracted the relative risk estimates of men and women separately whenever they were treated as independent samples. If authors reported different types of PA assessments, lifelong PA, vigorous PA, and quantitative PA were given priority in our assessments. When selecting quantitative PA assessments, we utilized PA frequency as this was the most common quantitative PA measurement component, and the frequency was reported as the number of PA periods per week.

In the 10 studies that we selected, four [23–26] reported relative risk estimates with high levels of PA as the reference group, and we converted the reported relative risk estimates (RRi) to reciprocal values. The remaining studies directly involved high PA.

### Study quality

Study quality was evaluated with the Newcastle–Ottawa Scale (NOS), with the evaluation carried out by two people independently (F.F.X. and L.Y.Y.). A third experienced manager joined the discussion and decided the outcome in case of any disparate evaluation results. The total score for the NOS of nine points was primarily used to evaluate case–control studies (exposure, selection, and comparability) and cohort studies (comparability, outcome, and selection). Quality scores lower than four were assigned to low-quality studies, and those ranging from five to nine were determined to be high-quality studies [27].

### Data analysis

The odds ratios and hazard ratios were interpreted to reflect the relative risk estimates (RRi) in measuring the association between PA and infertility risk. We directly extracted the most comprehensively adjusted risk estimates reported in the original literature when RRi values were available. Otherwise, we calculated RRs and 95% CIs via Stata software, version 11.0. We then calculated the relative risk estimate log (RRi) and its corresponding natural logarithm: S.E. si = (log [upper 95% CI limit of the RR] − log [RR])/1.96, and a random-effects model was exploited rather than a fixed-effects model to account for the weighted averages of the log (RRi)s, while taking into consideration the heterogeneity of effects measurements. In addition, the log (RRi)s by wi = 1/(si^2^ + t^2^) were weighted, where si represented the standard error of log (RRi), and t^2^ represented the restricted maximum likelihood estimate of the overall variance. Heterogeneity was obtained using Q- and I^2^-statistics [28]. We employed I^2^ statistics to assist in characterizing the heterogeneity between studies, and showed an I^2^ < 25%, which designated an important indicator of heterogeneity [28]. Considering that our results may be biased, we utilized funnel plots, Begg’s rank method [29], and Egger's linear regression method to detect publication bias [30]. p-values were considered to be statistically significant at the 0.05 level.

In a sub-analysis we examined the relation of PA to infertility risk with the classification of sex (men, women), study design (case–control, cohort), PA intensity (high, moderate and low), component or measure of PA (qualitative assessments, activity frequency), number of adjustment factors (smoking, marital status, weight or height), adjusted body mass index (BMI: yes, no), adjusted smoking (yes, no) and geographic region (United States of American (USA), United Kingdom (UK), and Asia).

Statistical analyses were conducted with Stata version 11.0 software (Stata Corp LP, College Station, TX, USA), and relative risk estimates were reported with 95% CIs. The statistical significance level of this study was 0.05 (α = 0.05).

## Results

**Fig. 1:**
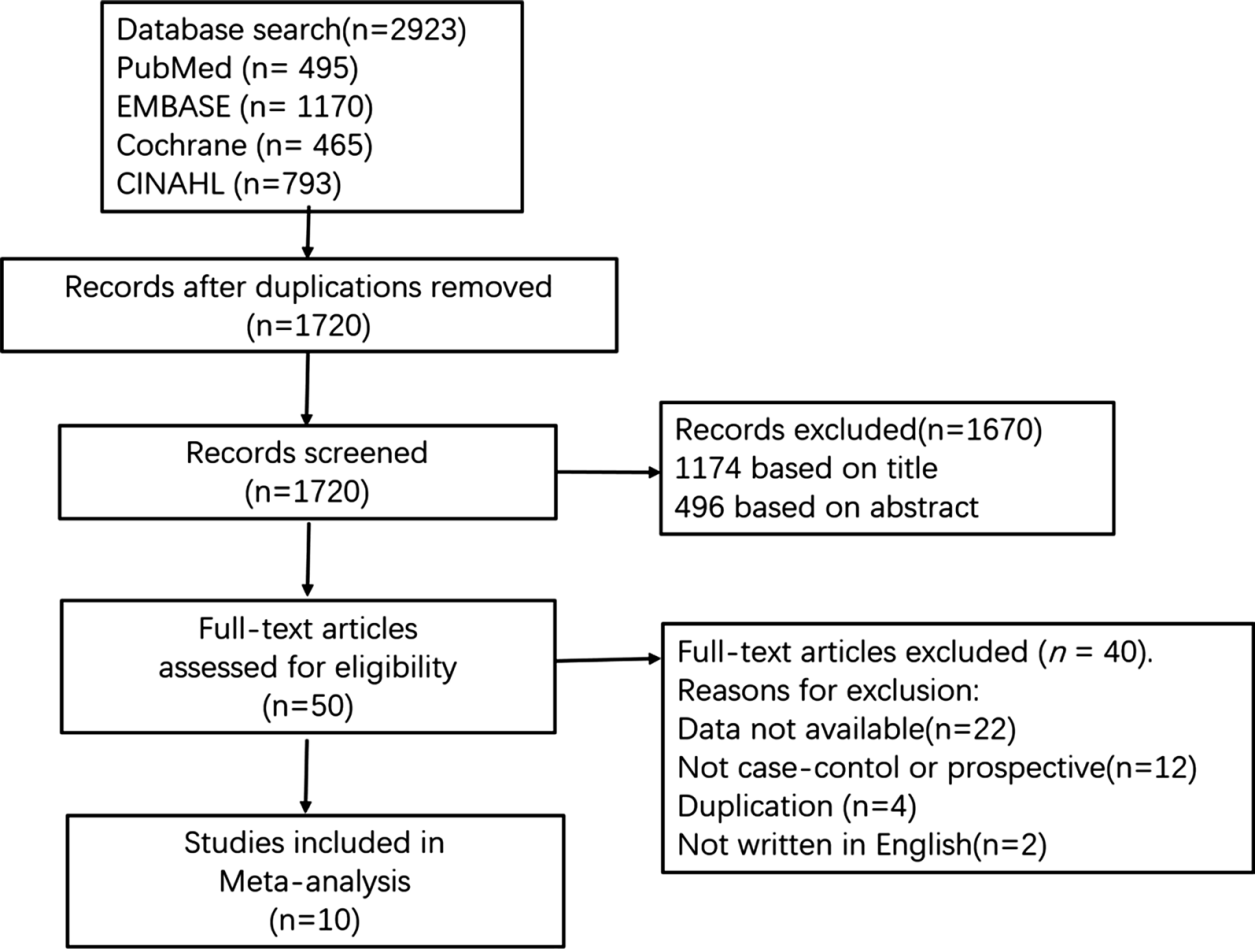
PRISMA flow diagram of identification and selection of eligible studies

A total of 708,965 subjects and 12,580 cases were included in this meta-analysis. We initially retrieved 2923 studies through four databases (495 from PubMed, 1170 form EMBASE, 465 from the Cochrane Library, and 793 from CINAHL), of which 1720 articles were retained after we removed duplicate studies. We also excluded unrelated studies by reading the titles and abstracts, and read the remaining 50 studies in depth; of these, 40 were further excluded because PA or relative risk date was not available or the study design did not meet our requirements. In one study the authors examined male and female samples separately and analyzed the risk of infertility in PA for men and women [23]. We then ultimately included in the present work 10 studies that yielded 12 different relative risk estimates [3, 21, 23–26, 31–34] (Fig. 1).

Table 1 shows the principal characteristics of the six cohort studies [3, 21, 24, 26, 31, 32] and four case–control studies [23, 25, 33, 34] of PA and infertility in our meta-analysis. The studies were conducted in eight countries: two in China [3, 31], two in the UK [32, 33], and the remaining six in the USA [34], Iran [24], Norway [21], Estonia [26], the Palestinian Territories [25], and France [23]. Two-thirds of the analyzed factors were adjusted for smoking, alcohol consumption; and obesity; and one-third of the risk estimates were adjusted for other factors such as age, marriage, medication use, and marital status. In addition, according to the five studies we included [23, 31–34], PA over 150 min/week was defined as high PA, less than 30 min as low PA, and 30–150 min as moderate PA (depicted in Table 1).

**Table 1.** Characteristics of the 8 studies on physical activity and digestive system cancer risk included in the meta-analysis

Authors, year	Gender	Region	Subjects	Cases	Relative Risk (95%CI) for high vs low PA	Relative Risk (95%CI) forhigh vs moderate PA	Relative Risk (95%CI) for moderate vs low PA	Low PA defined by	Moderate PA defined by	High PA defined by	Adjustment factors (excluding age ,sex)
Cohort studies											
Esmaeilzadeh et al. 2013	Women	Iran	2162	1081	0.77 (0.50, 1.19)	0.96 (0.56, 1.56)	0.80 (0.76, 0.83)	Low PA	Moderate PA	High PA	None
Lei et al. 2015	Women	China	367	310	0.38 (0.11, 1.29)	None	None	Non-exercise	None	≥ 180 min/week	Adjusted on occupational exposure, family income, menstruation,Chinese herbal medicine use, alcohol consumption
Cong et al. 2016	Women	China	670000	5131	0.58 (0.42, 0.81)	2.32 (1.25, 4.20)	0.25 (0.10, 0.65)	Light exercise	Regular PA	Heavy exercise	Adjusted for age at marriage, marriage age limit, weight andheight
Gudmundsdottir et al. 2009	Women	Norway	7774	3887	0.60 (0.30, 1.2)	1.67 (1.53, 1.67)	0.90 (0.60, 1.50)	Low PA	Medium PA	High PA	Adjusted for smoking and marital status
Rich-Edwards et al. 2002	Women	England	26125	830	0.63 (0.44, 0.91)	None	None	< 30 min/week	None	≥ 420 min/week	Adjusted for time spent in moderate activity, recency of oralcontraceptive use, intake of alcohol, and cigarette smoking
Läänelaid et al. 2021	MenWomen	Estonia	64 64	128	0.94 (0.66, 1.35) 0.43 (0.30, 0.62)	1.37 (1.33, 1.41) 1.11 (1.08, 1.13)	0.69 (0.49,0.96) 0.39 (0.27, 0.55)	Sedentary	Moderate PA	Vigorous PA	Adjusted for age and registered time.
Case-control studies											
Forman et al. 1994	Men	England	1587	794	0.54 (0.32, 0.90)	None	None	Non-exercise	None	≥ 900 min/week	Adjusted for undescended testis and inguinal hernia diagnosed < 15 years
Green et al. 1986	Women	American	200	100	0.90 (0.20, 3.60)	None	None	Non-exercise	None	≥ 150 min/week	Adjusted for race, census tract of residence, Reference year, parity, and times married.
Foucaut et al. 2019	MenWomen	French	151151	7980	0.45 (0.22, 0.94) 0.63 (0.29, 1.37)	None	None	< 150 min/week	None	≥ 150 min/week	Adjusted for educational level and for all variables of the table
Dhair et al. 2020	Women	Palestine	320	160	0.32 (0.17, 0.63)	0.75 (0.42, 1.35)	0.43 (0.40, 0.46)	Low PA	Moderate PA	High PA	None

**Fig. 2:**
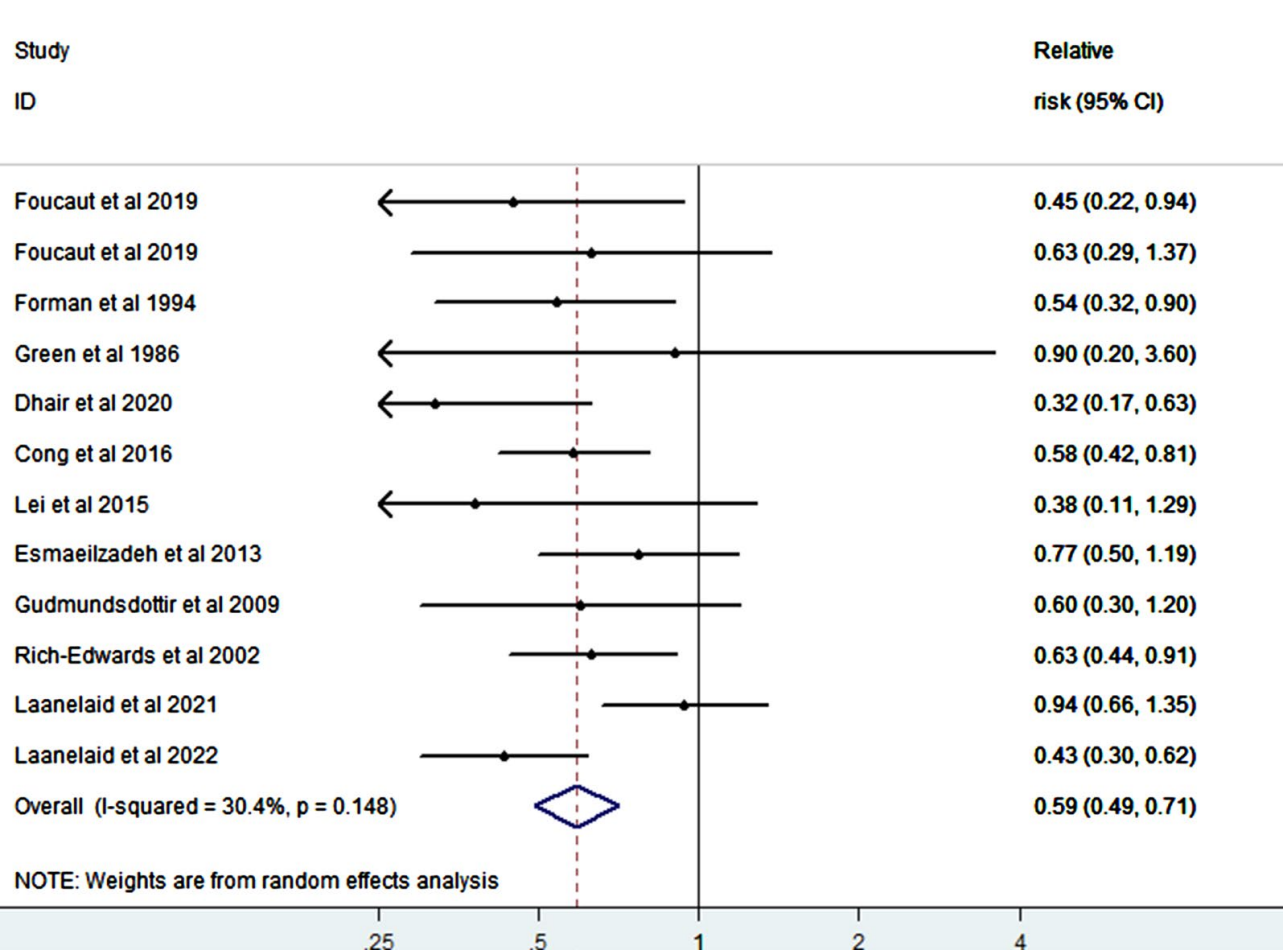
Forest plot of a random effects meta-analysis including 12 risk estimates of infertility for a high versus low level of PA

**Fig. 3:**
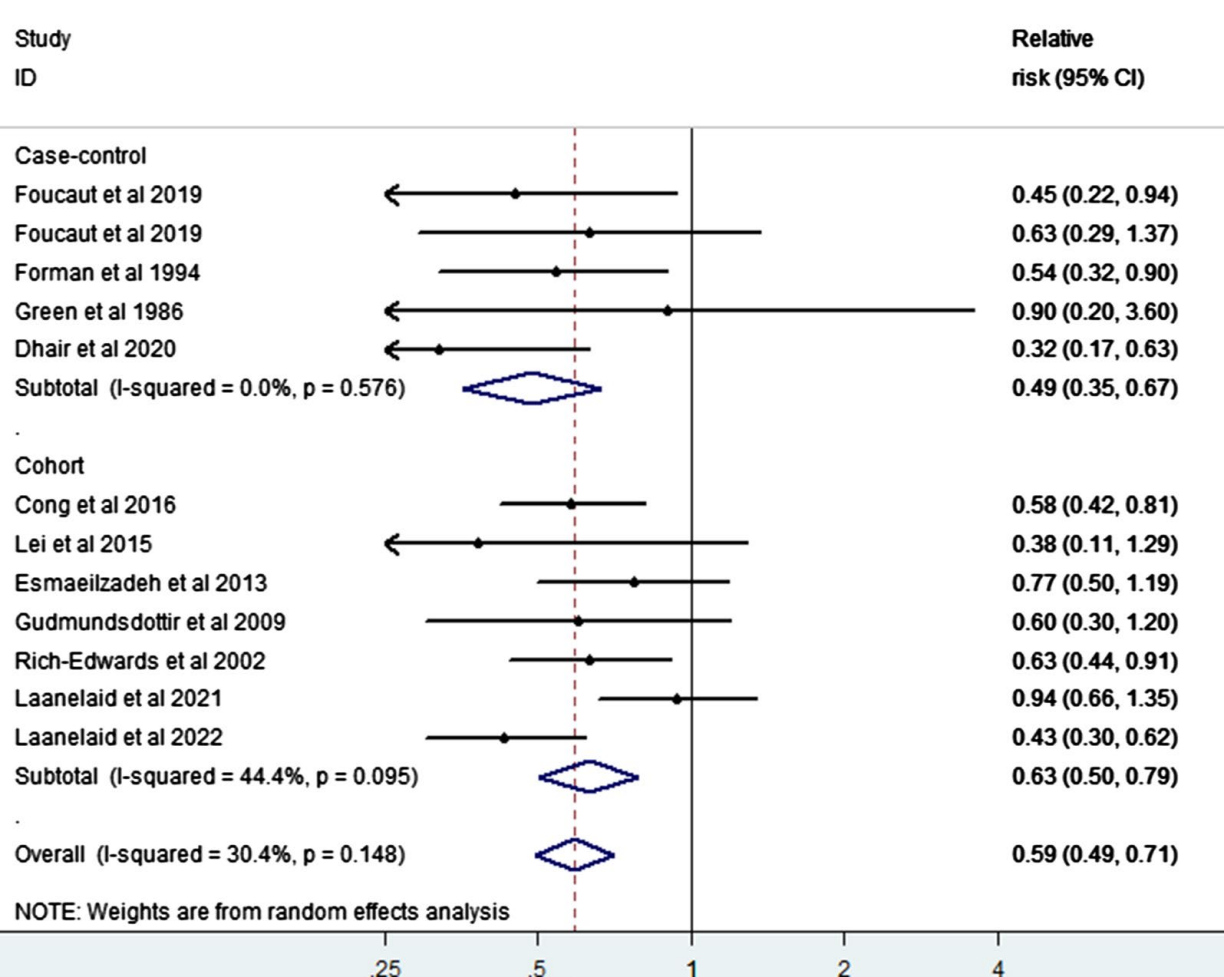
Forest plot of a random effects meta-analysis including 12 risk estimates of infertility for a high versus low level of PA, grouped by study design

**Fig. 4:**
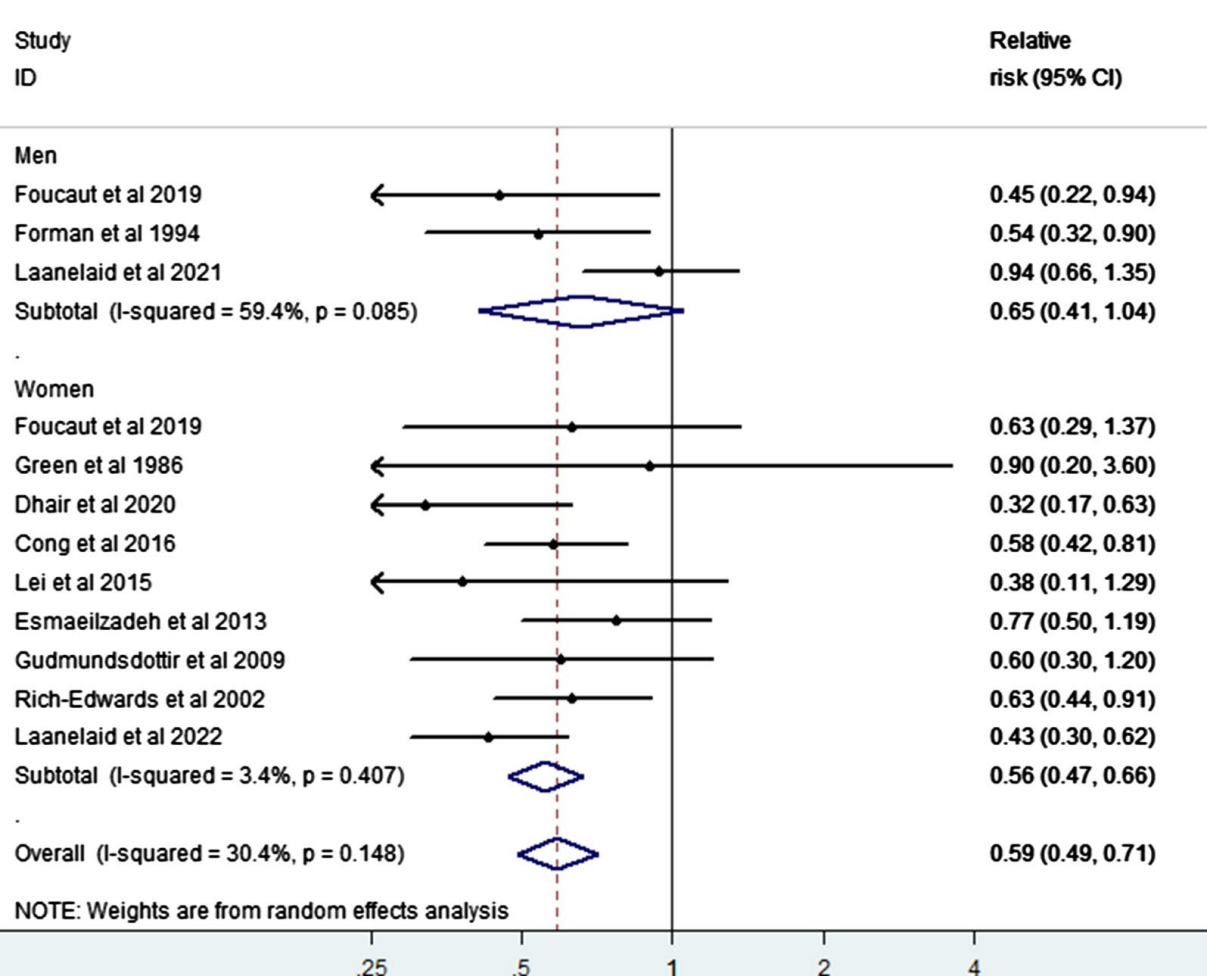
Forest plot of a random effects meta-analysis including 12 risk estimates of infertility for a high versus low level of PA, grouped by gender

We summarized 12 relative risk estimates using the random-effects model to reveal a 41% reduction in infertility risk with a high vs. low level of PA (RR = 0.59; 95% CI 0.49–0.71), and with low heterogeneity among studies (I^2^ = 30.4%, p-value for heterogeneity across all studies < 0.001) (Fig. 2). When we executed a stratified analysis of the study-design type, we observed a 37% reduction in infertility risk when a case–control study was removed (RR = 0.63; 95% CI 0.50–0.79, heterogeneity Chi-squared = 44.4%, p < 0.001). Similarly, when the cohort studies were removed, a 51% reduction was observed in infertility risk (RR = 0.49; 95% CI 0.35–0.67, heterogeneity Chi-squared = 0.0%, p < 0.001). The P-value for heterogeneity across the case–control and cohort studies were all significant (p ≤ 0.001) (Fig. 3). In addition, the pooled estimates from both the case–control and cohort studies showed low heterogeneity (I^2^ = 0%). When we analyzed our data according to the factor of sex, a direct comparison between women and men revealed a stronger inverse association between PA and infertility in men (RR = 0.65, 95% CI 0.41–1.04, p = 0.075) than women (RR = 0.56, 95% CI 0.47–0.66, p = 0.000) (Fig. 4).

**Fig. 5:**
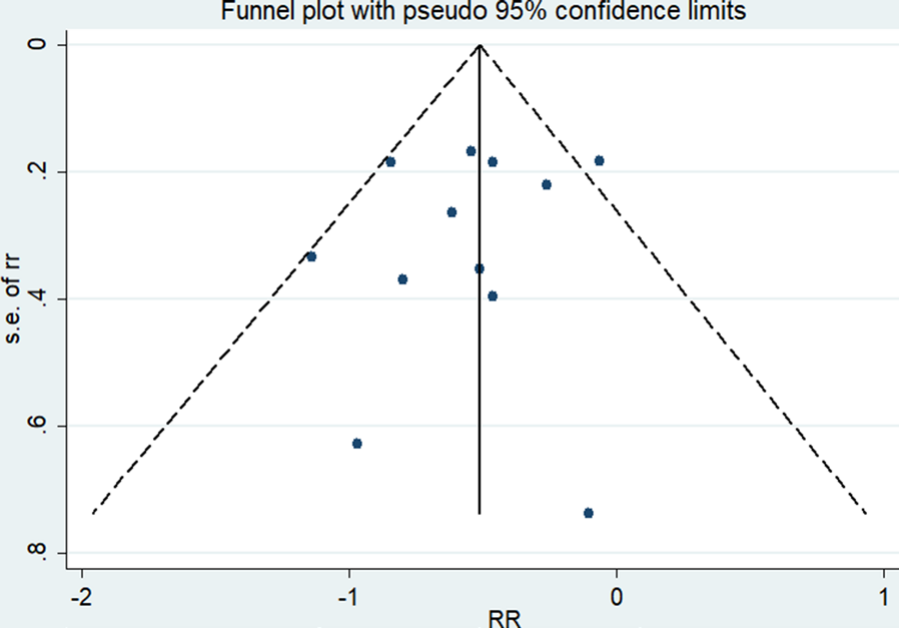
Standardized Funnel plot corresponding to the main random-effects meta-analysis

**Fig. 6:**
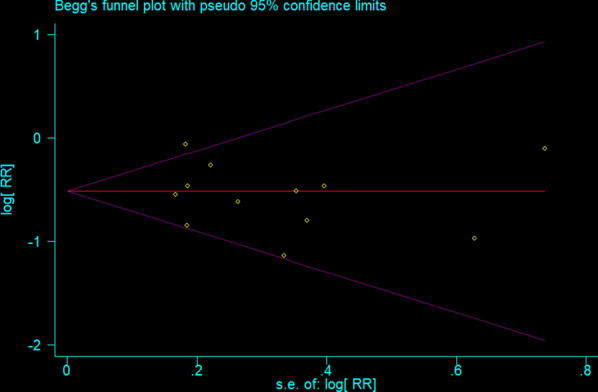
Standardized Begg’s rank correlation test corresponding to the main random-effects meta-analysis

**Fig. 7:**
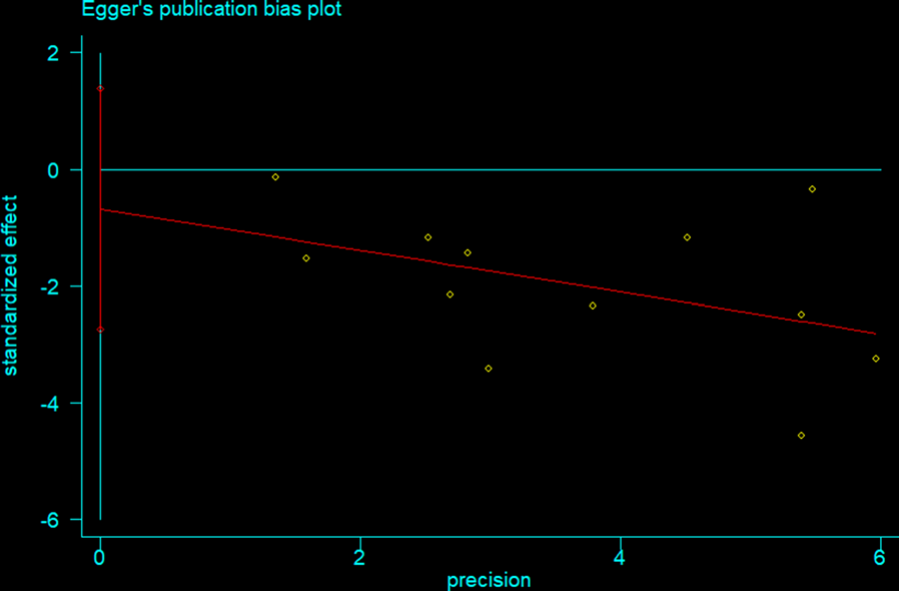
Standardised Egger’s regression test corresponding to the main random-effects meta-analysis

In order to determine publication bias, we constructed a funnel plot (Fig. 5), and executed Begg’s rank correlation test (p = 0.95) (Fig. 6) and Egger’s regression test (p = 0.48) (Fig. 7), with none indicating publication bias (p > 0.05).

**Fig. 8:**
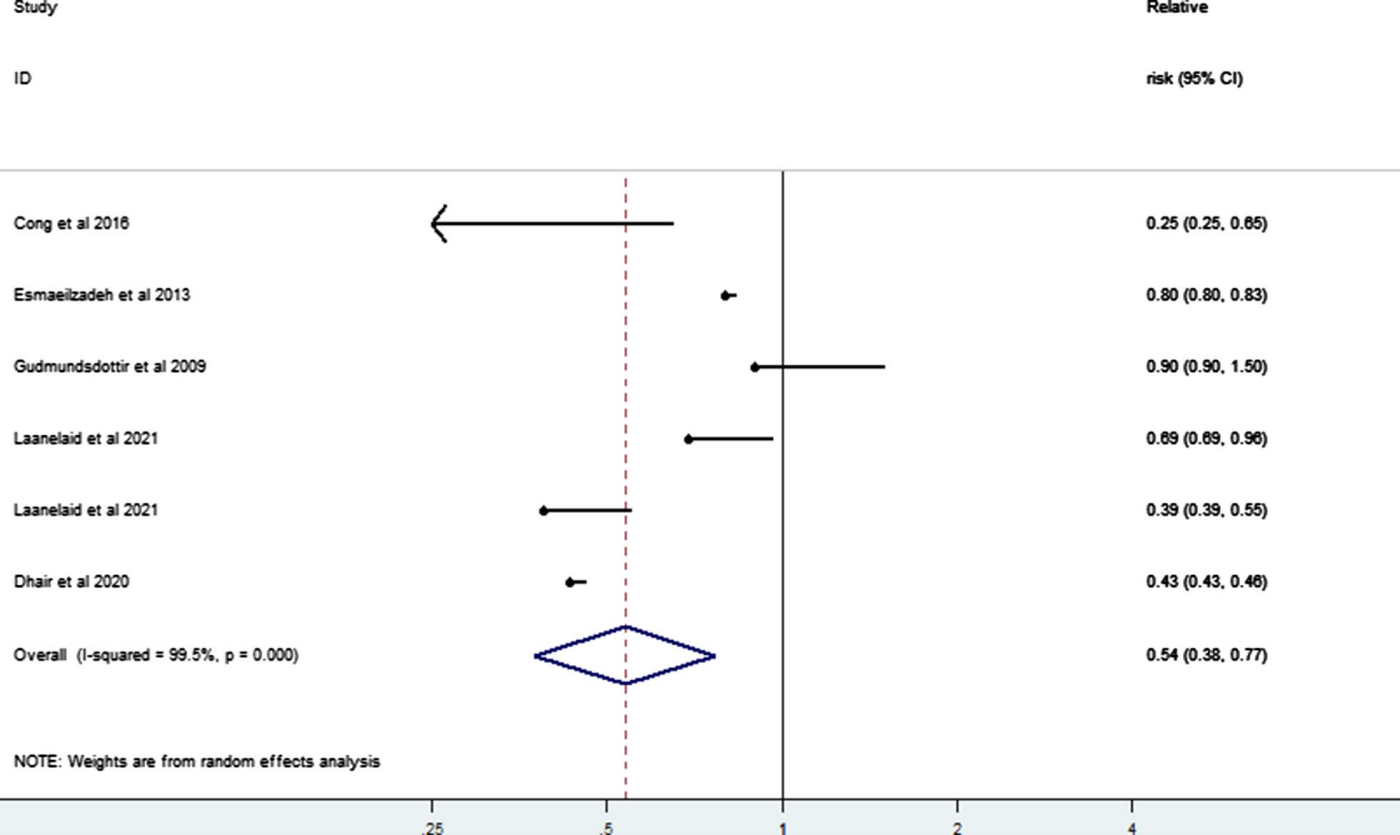
Forest plot of a random effects meta-analysis including 6 risk estimates of infertility for a moderate versus low level of PA

**Fig. 9:**
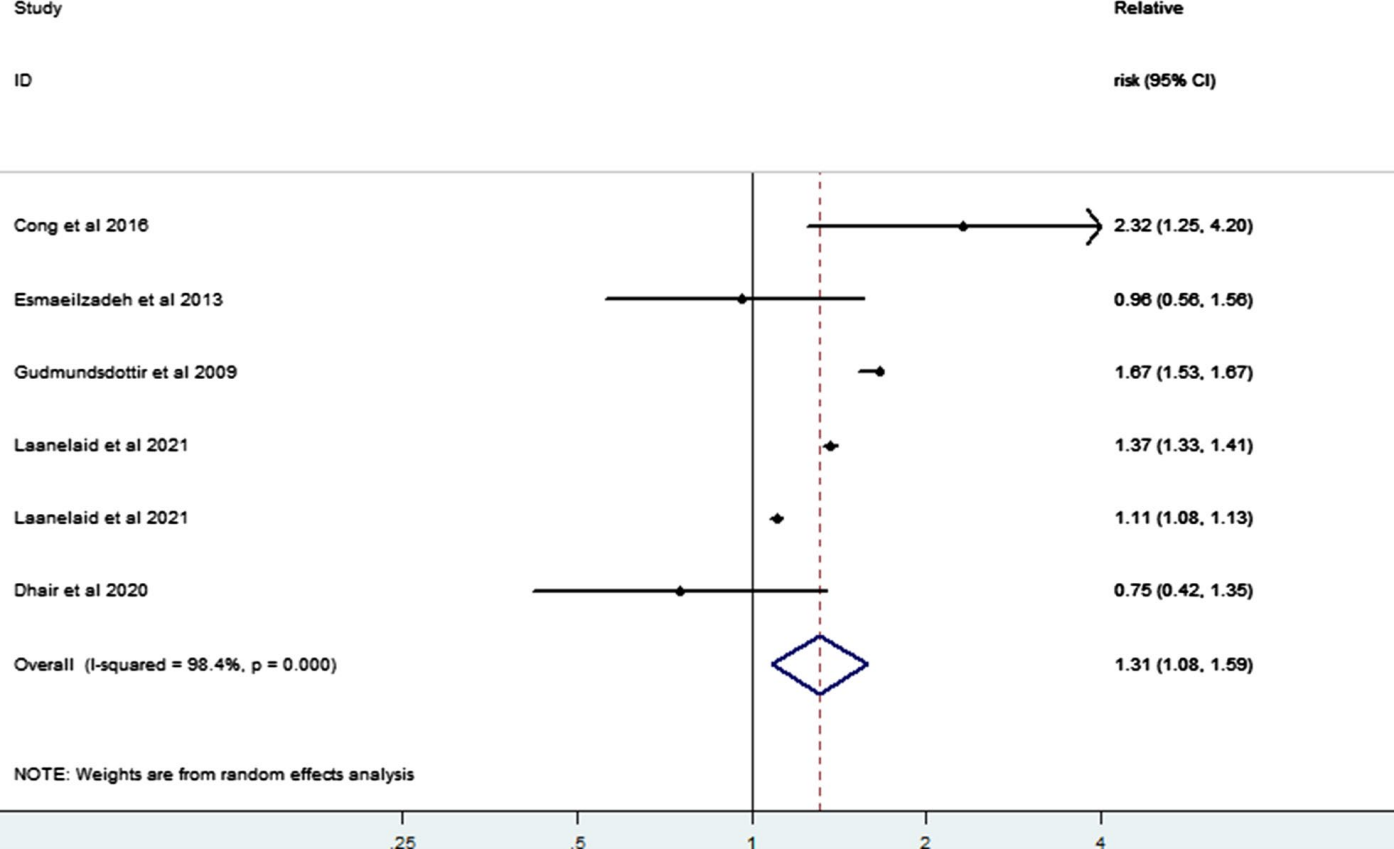
Forest plot of a random effects meta-analysis including 4 risk estimates of infertility for a high versus moderate level of PA

**Fig. 10:**
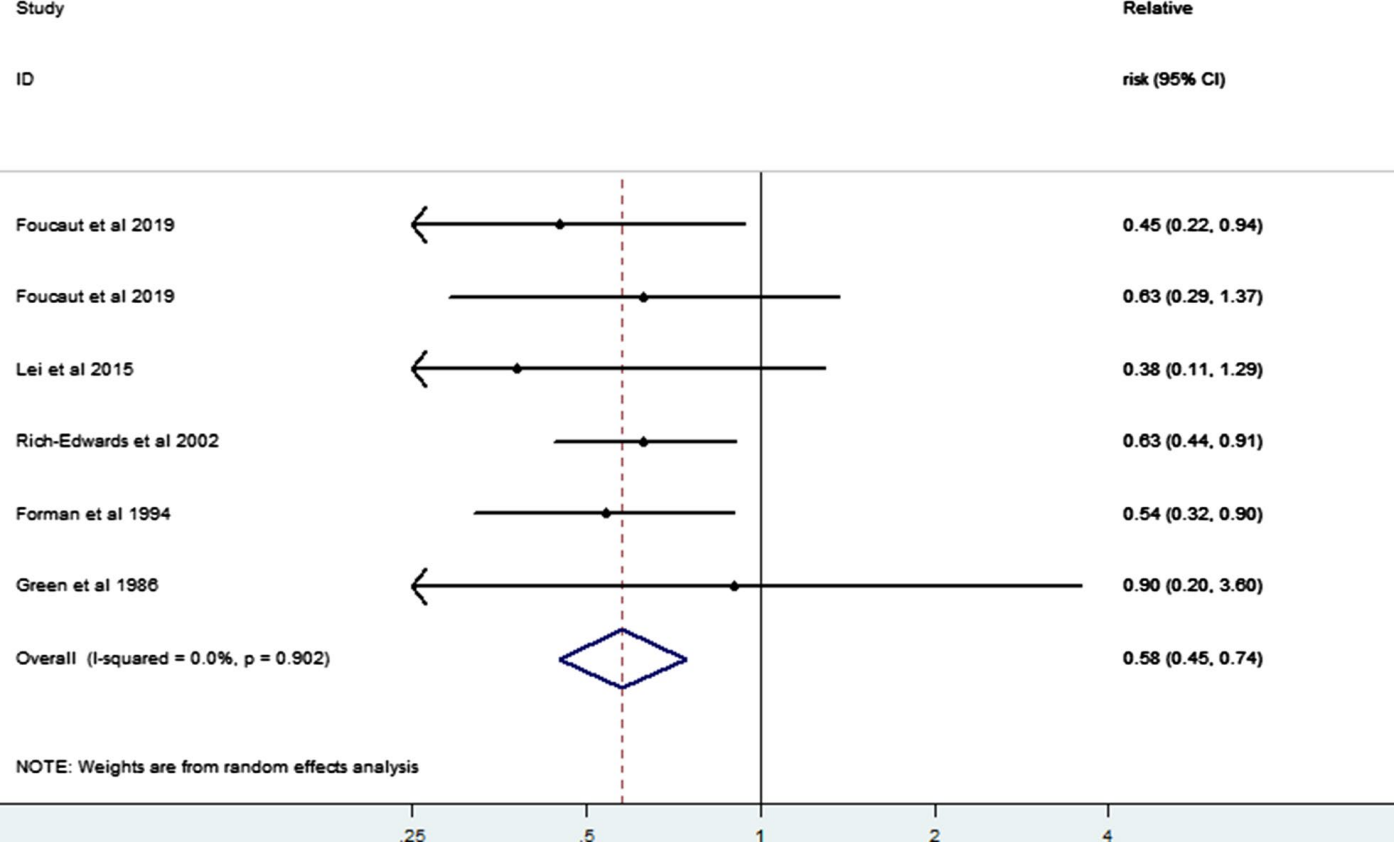
Forest plot of a random effects meta-analysis including 6 risk estimates of infertility for PA guidelines

Regarding the impact of differing levels of PA on the risk of infertility, our meta-analysis of six risk estimates from five studies [3, 21, 24–26] of low, moderate, and high PA levels showed that moderate PA may also reduce the risk of infertility compared with low PA (RR = 0.54, 95% CI 0.38–0.77) (Fig. 8); while compared with moderate PA, high PA increased the risk of infertility (RR = 1.31, 95% CI 1.08–1.59) (Fig. 9). In addition, our limited evidence [23, 31–34] suggested that compliance with international PA guidelines greatly lowered the risk of infertility (RR = 0.58, 95% CI 0.45–0.74; I^2^ = 0.0%) (Fig. 10).

An evaluation of study quality is depicted in Table 2, with an average quality score of 6.10 (SD = 0.57, median = 6). The studies we included contained a variety of potential confounders, including smoking (2/8), drinking alcohol (2/8), and marital status (2/8), with none of the studies showing a correction of infertility symptoms. Among these studies, investigators in four studies diagnosed and defined infertility clearly, and the overall evaluation of our included articles was above six points. The studies reflected high-quality research according to the questionnaire survey, although they did not indicate whether their case group was a continuous case or acknowledge its representativeness.

**Table 2:** Quality of studies according to Newcastle-Ottawa Scale

First author, Year, country	Selection (Max,score4)	Comparability (Max,score2)	Exposure(case-control) or outcome (cohort) (max,score3)
Cohort			
Rich-Edwards, 2002	2	2	2
Cong, 2016	2	2	1
Esmaeilzadeh, 2013	2	2	2
Lei, 2015	3	1	2
Gudmundsdottir, 2009	4	1	2
Läänelaid, 2021	3	2	1
Case-control			
Foucaut, 2019	4	1	2
Green, 1986	3	2	1
Forman, 1994	3	1	2
Dhair, 2020	3	2	1

## Discussion

PA fosters development and normal growth, improves mood, function, and sleep quality, and lowers the risk of chronic diseases [35, 36]. In the field of reproduction, new WHO guidelines recommend that pregnant women achieve at least 150 min/week of vigorous-intensity aerobic exercise to help increase their chances of becoming pregnant, and to also improve their overall health [16]. These recommendations are considered safe in the treatment of infertility and are consistent with ours. In addition, the WHO Guidelines Advisory Committee graded the evidence based on the consistency and quality of the research, with evidence graded as strong or moderate used as the basis for the key guidelines [37, 38]. This was reflected in our results with regard to high-to-moderate PA. In 2018, the American College of Sports Medicine International Multidisciplinary Roundtable was tasked with updating the recommendations and focused on evidence from the exercise method, including resistance exercise and aerobic exercise [39]. Thus, in our meta-analysis we selected exercise patterns based on differing levels of PA; i.e., high vs. low PA, high vs. moderate PA, and moderate vs. low PA—as well as on meeting vs. not meeting the international PA guidelines. We implemented a random-effects meta-analysis on the association between PA and infertility, and our results indicated that PA was a protective factor against infertility; and that compared with low PA, high PA levels reduced the risk of infertility by 39% and moderate PA did so by 30%. However, compared to moderate PA, high PA increased the risk of infertility (RR = 1.31, 95% CI 1.08–1.59).

An analysis stratified by sex and research types showed that PA exerted a protective effect on infertility, and that the summary RR estimate was not affected by individual and potentially influential factors such as sex, study region, study design, or the number of adjustment factors. The effects of individual factors were verified in previous meta-analyses of PA and polycystic ovary syndrome [22]**,** reproductive health [40], and depression [41]**.** Congruent with our results, these reports showed no statistically significant heterogeneity across sex [41]**,** study design [22, 40], geographic region [42], or adjustment factors [40].

In 2016, the authors of a review explored the impact of PA on infertility patients [43]. Several methodologic issues related to PA and infertility were discussed, including a definition of the components of PA, assessment of the research (e.g., type, duration, and frequency), and a consideration of the optimal way to measure PA. Investigators evaluated several influencing factors and eventually concluded that PA may alleviate infertility through variables such as body mass index and age [44]. Another study showed that when obese infertile women increased their PA, such lifestyle changes not only improved their fertility, but also improved the overall health of their offspring [45]. Another paper focused on the relationship between PA and endometriosis risk in women with infertility or pain, and revealed that PA reduced pain and infertility caused by endometriosis; however, the authors left as unexplained whether their findings could be interpreted as constituting a role for exercise at the molecular and endocrine levels, or whether the findings were related to a variety of complex mechanisms such as study design, control selection, and PA in improving infertility. There are currently no data as to the potential effects of PA on infertility.

A recent study in which the efficacy of PA on pregnancy rate [46] focused on PA intensity included both moderate and vigorous intensity. Herein we finally concluded that any amount of vigorous PA was associated with increased pregnancy rate and that pregnant patients need to be supported in their exercise regimens during pregnancy. Our findings on the impact of PA on infertility are thus virtually identical to the results of the previous study.

Several mechanisms may explain our current findings. PA in women experiencing infertility may lead to the resumption of ovulation by regulating the hypothalamic–pituitary–adrenal (HPA) axis so as to increase hypothalamus-pituitary–gonadal (HPG) activity. High PA with energy consumption and opioid fluctuations caused by excessive exercise also demonstrated HPA dysfunction. Long-term exercise for women with infertility can decrease insulin and free androgen levels, leading to HPA mediating the recovery of infertility [47]. Other underlying mechanisms of infertility have been explained as related to toxic metals causing adverse reproductive effects. For example, non-essential metals that include cadmium (Cd), lead (Pb), and arsenic (As) are reproductive toxicants that are widely distributed within the environment and affect hormonal levels. Infertile women may manifest reduced sensitivity by their ovaries to gonadotropins, resulting in higher circulating gonadotropin levels such as higher average serum FSH and LH levels, and high serum FSH levels indicate poor ovarian function [48]. Several studies have shown that engaging in PA might assist in reducing Pb accumulation and gonadotropin levels in infertile women [31, 49–51]. In addition to these mechanisms subserving the influence of PA on infertility, PA is also known to activate anti-oxidant defenses and increase immune function. PA significantly inhibited inflammatory biomarkers (interleukin-6 and tumor necrosis factor-α), oxidative stress (reactive oxygen species and malondialdehyde), and antioxidants (superoxide dismutase, catalase, and total antioxidant capacity); and these changes coincided with favorable improvements in semen parameters, sperm DNA integrity, and pregnancy rate. These data indicated that PA was sufficient in improving male reproductive function markers in infertile patients [52].

One novel aspect of the current study was the use of known varieties of infertility to conduct a meta-analysis of the relationship between PA and infertility. Another strength of the current study was a minimized publication bias in our systematic exploration of databases in the fields of infertility and PA. We categorized the studies by design, conducted subgroup analyses for each design group, and implemented a tool for the assessment of study quality to address potential selection, misclassification, and confounder biases specifically. We ultimately uncovered an inverse association between PA and infertility risk in analyses that included all risk estimates from high-quality studies.

## Conclusion

Our comprehensive meta-analysis provided support for an inverse relationship between PA and risk of infertility, revealing that a moderate to high PA level significantly reduced the overall risk of infertility and was a common protective factor. In addition, limited evidence suggested that compliance with international PA guidelines greatly lowered the risk of infertility (RR = 0.58, 95% CI 0.45–0.74; I^2^ = 0.0%). In the future, investigators need to determine the frequency, optimal dosage, and duration of PA required to effectively reduce the risk of infertility.

## Supplementary Information

**Additional file 1**: Proof of polishing changes to the language

**Additional file 2**: Screenshot of registration link

## Data Availability

All data generated are included in this published article.

## Abbreviations

PA: Physical activity
RR: Summary relative risk
CI: Confidence interval
CBT: Cognitive behavior therapy
WHO: World Health Organization
NOS: Newcastle–Ottawa Scale
BMI: Body mass index
HPA: Hypothalamic–pituitary–adrenal
HPG: Hypothalamus-pituitary-gonad
