# Supplementary material for: Association between physical activity and infertility: a comprehensive systematic review and meta-analysis
Source: J Transl Med. 2022 May 23;20:237. doi: 10.1186/s12967-022-03426-3 (PMC9125843; doi:10.1186/s12967-022-03426-3)
Supplement: Supplementary file 1 — Additional file 1: Proof of polishing changes to the language [file 12967_2022_3426_MOESM1_ESM.pdf]

Certificate of English Language Editing

Manuscript Title:

Association between physical activity and infertility: a comprehensive systematic review and meta-analysis

Date of Revision:

April 21, 2022

Abstract:

Background: Physical activity (PA) may protect against infertility by modulating the hypothalamic-pituitary-gonadal axis, thereby reducing gonadotropin levels, elevating immune function, and inhibiting inflammation and circulating sex hormones. However, whether PA reduces the risk of infertility remains largely unknown. We therefore conducted a systematic review and meta-analysis to determine the preventive effects of PA on infertility.

Methods: We searched PubMed, Cochrane Library, EMBASE, and CINAHL databases to retrieve published epidemiologic studies on the relationship between PA and infertility. Following the PRISMA guidelines, we selected English literature published prior to 11 April 2022, and assessed study quality using the Newcastle-Ottawa Scale. Our protocol, including the full methods employed for this review, is available on PROSPERO (ID=CRD42020143344).

Results: Six cohort studies and four case-control studies based on 708,965 subjects and 12,580 cases ...

This document certifies that the manuscript listed above was copy edited for English language by LetPub, with regard to grammar, punctuation, spelling, and clarity. All of our language editors are native English speakers with long-term experience in editing scientific and technical manuscripts. We are committed to leveling the playing field for researchers whose native language is not English.

- Documents receiving this certification should be regarded as having undergone professional editorial revision for English language before submission. However, the authors may accept or reject LetPub's suggestions and changes at their own discretion and LetPub does not have editorial control over the submitted documents.
- The language quality of the submitted document is the sole responsibility of the submitting authors subject to those authors' adherence to LetPub's revisions and instruction. LetPub's provision of service does not constitute a guarantee or endorsement of the authors' work herein.
- Neither the research content nor the authors' intended meaning were altered in any way during the editing process.
- If you have any questions or concerns about this edited document, please contact us at support@letpub.com

LetPub
Your Manuscript Editor

LetPub is an author service brand owned and operated by Accdon LLC. Headquartered in the Boston area, we are a full-spectrum author services company with a large team of US-based certified language and scientific editors, ISO 17001 accredited translators, and professional scientific illustrators and animators. We advocate ethical publication practices and are an official member of the Committee on Publication Ethics (COPE).

For more information about our company, services, and partnership programs, please visit www.letpub.com.

© 2022 Accdon, LLC. All Rights Reserved. Tel: 1-781-202-9968 Email: info@accdon.com Address: 400 Fifth Ave, Suite 530, Waltham, MA 02451, United States
