# Supplementary figures and images for: Association between physical activity and infertility: a comprehensive systematic review and meta-analysis
Source: J Transl Med. 2022 May 23;20:237. doi: 10.1186/s12967-022-03426-3 (PMC9125843; doi:10.1186/s12967-022-03426-3)

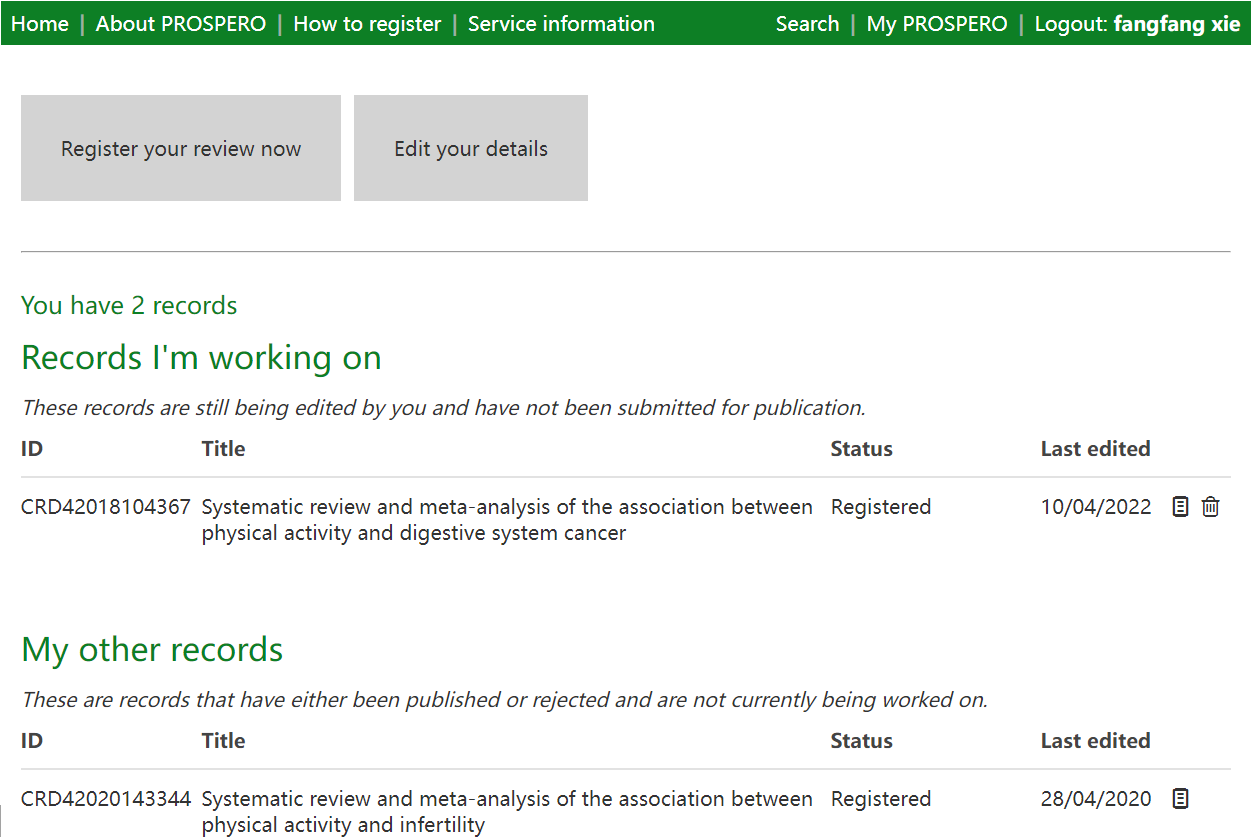

Supplement: Supplementary file 2 — Additional file 2: Screenshot of registration link [file 12967_2022_3426_MOESM2_ESM.png]
